# The prevalence of metabolic syndrome in chronic obstructive pulmonary disease: A systematic review and meta-analysis

**DOI:** 10.1177/14799731251346194

**Published:** 2025-05-26

**Authors:** Hassan Alrabbaie, Mohammad Al-Wardat, Mohammad Etoom, Marla Beauchamp, Roger Goldstein, Dina Brooks

**Affiliations:** 1 School of Rehabilitation Sciences, Faculty of Health Science, McMaster University, Hamilton, ON, Canada; 2 Department of Rehabilitation Sciences, Faculty of Applied Medical Sciences, 37251 Jordan University of Science and Technology, Irbid, Jordan; 3 Department of Respiratory Medicine, 27375 West Park Healthcare Centre, Toronto, Canada; 4 Department of Physical Therapy, Faculty of Medicine, University of Toronto, Toronto, ON, Canada; 5 Rehabilitation Sciences Institute, School of Graduate Studies, University of Toronto, Toronto, ON, Canada

**Keywords:** COPD, metabolic syndrome, prevalence, diagnostic criteria, GOLD stages

## Abstract

**Objective:**

Metabolic syndrome (MetS) is a cluster of factors that increase the risk of cardiovascular disease and type 2 diabetes. It is highly prevalent among patients with Chronic Obstructive Pulmonary Disease (COPD). This systematic review and meta-analysis assessed MetS prevalence in COPD patients, focusing on variations by gender, diagnostic criteria, and disease severity.

**Methods:**

We systematically searched MEDLINE, Embase, Scopus, and CINAHL. Two reviewers independently extracted data using a standardized form, and study quality was assessed with the Joanna Briggs Institute checklist. Prevalence rates, with 95% confidence intervals (CI), were calculated using a random-effects model. Subgroup analyses by sex, COPD severity, and MetS components were conducted.

**Results:**

Forty-two studies, including 54,278 COPD patients, were analyzed. Overall, the prevalence of MetS was 37% (95% CI: 30.6–43.8%; I^2^ = 99.03%, p < 0.001). Prevalence was 48% (95% CI 38.1 to 57.5) in males and 43% (95% CI 38.3 to 48.8) in females. Among studies using the Alberti definition, the pooled prevalence was 46% (95% CI 35.6 to 56.3). Patients with GOLD stage II showed a prevalence of 44% (95% CI 37.3 to 50.4). The most common MetS components were Hypertension 58% (95% CI 47.2 to 68.0) and increased waist circumference 51% (95% CI 37.1 to 64.6).

**Conclusion:**

MetS is highly prevalent among COPD patients. Standardized diagnostic criteria are needed, and early detection with integrated care is recommended.

## Introduction

Chronic Obstructive Pulmonary Disease (COPD) is a widespread and progressive respiratory condition characterized by persistent airflow limitation and chronic airway inflammation.^1^ COPD has a systemic implication that extends beyond the lungs, including Metabolic Syndrome (MetS), elevated risk of cardiovascular diseases, anxiety, depression, and anemia, leading to multimorbidity.^2–5^ It is a leading cause of morbidity and mortality worldwide and is currently among the top three causes of death globally.^6^ By 2030, the disease burden of COPD is expected to increase further, solidifying its position as one of the leading causes of death worldwide.^6,7^ Cardiovascular disease, type 2 diabetes, and hypertension are commonly observed across all stages of COPD, as defined by the Global Initiative for Chronic Obstructive Lung Disease Stages (GOLD),^8^ influencing mortality and hospitalization rates regardless of the severity of airflow obstruction.^9^

Metabolic Syndrome (MetS) is also prevalent in COPD patients.^10^ MetS is characterized by a collection of metabolic abnormalities, including central obesity, insulin resistance, hypertension, and dyslipidemia.^11–18^ These factors collectively elevate the risk of developing atherosclerotic, cardiovascular diseases, and type 2 diabetes mellitus (T2DM).^19^ A diagnosis of MetS requires the presence of at least three specified factors, with multiple definitions establishing specific thresholds for its components, as summarized in Table 1. Obesity is observed in approximately 18% to 22 % of patients with COPD and is significantly more prevalent in the early stages of the disease.^20,21^ In a study by García-Olmos et al., which included 198,670 participants over the age of 40, 3.2% were diagnosed with COPD. Among these patients, 20% had diabetes, 25% were obese, and 34% suffered from dyslipidemia.^22^ Importantly, obesity can significantly affect respiratory function, regardless of a COPD diagnosis.^23,24^

**Table 1.** Diagnostic Criteria for Metabolic Syndrome (MetS) in adults.

World health organization criteria (1998)^11^	European group for the study of insulin resistance criteria (1999) EGIR^12^	National cholesterol education program adult treatment panel III (NCEP: ATPIII) criteria (2005 and 2001)^13,104^	American association of clinical endocrinology (AACE)^15^	International diabetes federation (IDF) criteria (2005)^16^	American heart association/National heart, lung, and blood institute (AHA/NHLBI) criteria (2004)^17^	Alberti (2009)^18^
• Insulin resistance is defined as T2DM or IFG > 100 mg/dl) or IGT, plus two of the following	• Insulin resistance is defined as insulin levels > 75th percentile of non-diabetic patients, plus two of the following:	Any three or more of the following:	IGT plus two or more of the following:	Central obesity (defined as waist circumference ≥ 94 cm (Men), ≥80 cm (Women) but can be assumed if BMI > 30 kg/m2) with ethnicity-specific values*, plus two of the following:	Any three of the following:	At least three of five of the following
1. Abdominal obesity (waist-to-hip ratio > 0.9 in men or > 0.85 in women) or BMI > 30 kg/m2. 2. TG 150 mg/dl or greater, and/or HDL -Cholesterol < 40 mg/dl in men and < 50 mg/dl in women. 3. BP 4. 140/90 mmHg or greater. 5. Microalbuminuria (urinary albumin secretion rate 20 μg/min or greater, or albumin-to-creatinine ratio 30 mg/g or greater).	1. Waist circumference 94 cm or greater in men, 80 cm or greater in women. 2. TG 150 mg/dl or greater and/or HDL-cholesterol < 39 mg/dl in men or women. 3. BP 140/90 mmHg or greater or Rx 4. FG 110 mg/dl or greater.	1. Waist circumference > 102 cm in men, > 88 cm in women. 2. TG 150 mg/dl or greater. or Rx 3. HDL-cholesterol < 40 mg/dl in men and < 50 mg/dl in women. Or Rx 4. BP >130 mmHg systolic or >85 diastolic mmHg. or Rx 5. FG 110 mg/dl* or greater. Or Rx	1. BMI 25 kg/m2 or greater. 2. TG 150 mg/dl or greater and/or HDL- cholesterol < 40 mg/dl in men and < 50 mg/dl in women. 3. BP 130/85 mmHg or greater 4. FG between 110 and 126 mg/dL	1. TG 150 mg/dl or greater. Or Rx 2. HDL-Cholesterol < 40 mg/dl in men and < 50 mg/dl in women. or Rx 3. BP 130/85 mmHg or greater. Or Rx 4. FG 100 mg/dl or greater.	1. Waist circumference 102 cm or greater in men, 88 cm or greater in women. 2. TG 150 mg/dl or greater. HDL-Cholesterol < 40 mg/dl in men and < 50 mg/dl in women. 3. BP 130/85 mmHg or greater. 4. FG 100 mg/dl or greater	1. Waist circumference > 102 cm in men, and > 88 cm in women, with ethnicity-specific values for other groups. 2. FG ≥100 mg/dL or Rx 3. TG 150 mg/dl or greater or Rx 4. HDL-Cholesterol < 40 mg/dl in men and < 50 mg/dl in women or Rx 5. Systolic ≥130 and/or diastolic ≥85 mm Hg or Rx
			Other risk factors: family history of T2MD, hypertension, or CVD, polycystic ovary syndrome, sedentary lifestyle, advanced age. The ethnic group having a high risk for T2DM or CVD		To meet the criteria, waist circumference must be for Europeans, > 94 cm in men and > 80 cm in women; and for South Asians, Chinese, and Japanese, > 90 cm in men and > 80 cm in women. For ethnic South and Central Americans, South Asian data are used, and for sub-Saharan Africans and Eastern Mediterranean and Middle East (Arab) populations, European data are used.	

ABBREVEATION: IFG; Impaired Fasting Glucose, IGT; Impaired Glucose Tolerance, FG; Fasting Glucose, BMI; Body Mass Index, TG; Triglycerides, HDL; High-Density Lipoprotein, PB; Blood pressure, Rx; pharmacologic treatment, CVD; Cardiovascular Disease, T2DM; Type 2 Diabetes Mellitus.

The CONSISTE study conducted by Pilar de Lucas-Ramos et al. assessed cardiovascular risk factors in COPD patients and found a higher prevalence of ischemic heart disease (IHD) at 12.5% compared to 4.7% in controls.^25^ Additionally, dyslipidemia was present in 48.3% of COPD patients versus 32% in controls.^25^ Similarly, Mitra et al. found that COPD patients had a higher level of total cholesterol, Triglyceride (TG), and low-density lipoprotein (LDL) levels compared to the controls.^26^ Furthermore, evidence suggests that the prevalence of diabetes among patients with COPD typically ranges from 3% to 12%.^27,28^ Reduced lung function is a significant risk factor for the development of diabetes, particularly in patients with COPD in GOLD stages III and IV.^29,30^ The incidence of hypertension among COPD patients varies from 6% to 53 %, depending on disease severity.^31^ Globally, the prevalence of MetS is approximately 31 %,^32^ with rates of 38-42% in the USA,^33^ 15 % in Canada,^34^ and 21%−31% in Asia.^35,36^ This prevalence tends to increase with age and obesity,^37^ However, the prevalence of MetS, specifically among patients with COPD, remains uncertain, as studies often report inconsistent findings due to variations in diagnostic criteria and study designs.^38–41^ Several studies have reported considerable variability in the prevalence of MetS among COPD patients, ranging from 10% to 62%.^38,39,42–44^ For instance, a study applying the National Cholesterol Education Program Adult Treatment Panel III (NCEP-ATP III) criteria reported a prevalence of approximately 10% among COPD patients.^39^ In contrast, another study using the International Diabetes Federation (IDF) criteria found a much higher prevalence of around 60 %.^45^ Additional studies have reported prevalence rates falling within this broad range, depending on the diagnostic framework and population characteristics.^42,43,46,47^ These discrepancies demonstrate how the choice of diagnostic criteria directly affects prevalence estimates, contributing to the inconsistent findings observed in the literature.

This review systematically analyzed existing literature to assess the prevalence of MetS among patients with COPD. Additionally, we investigated the prevalence of individual MetS components and general clinical characteristics such as age, sex, BMI, and disease severity.

## Methods

This systematic review followed the Preferred Reporting Items for Systematic Reviews and Meta-Analyses (PRISMA) guidelines. The protocol was registered in the PROSPERO database (registration ID: CRD42024548580).

### Search strategy and study selection

A comprehensive literature search was conducted in consultation with a librarian across four databases: MEDLINE, CINAHL, Embase, and Scopus, covering publications from the year 2000 up to June 24, 2024. The starting point of year 2000 was chosen to ensure a focus on contemporary diagnostic criteria for MetS, which have evolved considerably following the introduction of standardized definitions. The research included the following key terms: chronic obstructive pulmonary disease (MeSH), metabolic syndrome (MeSH), and prevalence (MeSH). A detailed search strategy for each database is available in Supplemental Table S1. Additionally, two authors (H-A, MA-W) conducted a manual search to identify potentially relevant studies that were not captured by the database search terms.

### Eligibility criteria

This review included studies that reported the prevalence of metabolic syndrome (MetS) in cohorts of patients diagnosed with COPD. Studies were eligible if they clearly defined both COPD and MetS and provided distinct prevalence data for COPD patients. Studies were excluded if they failed to report results for COPD patients separately from the general sample, lacked a definition of MetS, or conflated COPD with other comorbidities without providing stratified data.

To ensure diagnostic consistency, we included studies that defined COPD based on spirometry-confirmed diagnosis in accordance with the GOLD criteria or equivalent standards.

Eligible study designs included case-control, cohort, cross-sectional, and interventional studies that reported the prevalence of MetS as a comorbidity in COPD patients. Excluded study designs included qualitative studies, case reports, case series, animal studies, and laboratory-based studies, as these do not provide quantitative prevalence estimates or do not apply to the human population. Additionally, non-peer-reviewed or non-primary research sources, such as conference proceedings, magazines, news articles, electronic resources, reports, theses, dissertations, abstracts, editorials, and systematic reviews were excluded from the analysis. However, the reference lists of these sources were screened for potentially relevant studies.

### Data extraction

Two independent reviewers conducted the data extraction process using a standardized data collection sheet designed for this review. This sheet captured essential information from each study, including study design, sample size, participant characteristics (age, sex, BMI, and severity of COPD), and the primary outcome of interest: the prevalence of MetS and its components (waist circumference, blood glucose, blood pressure, triglycerides, high-density lipoprotein) (see Supplemental Table S3).

### Assessment of study quality

Two independent authors (H-A and MA-W) assess the quality of each study using the Joanna Briggs Institute (JBI) critical appraisal checklist.^48^ JBI’s tool evaluates various domains of study design and execution, including the appropriateness of the sample frame, recruitment methods, sample size adequacy, clarity of subject and setting description, the rigor of analysis, and the reliability of measurement tools.

The reviewers assessed each study across nine criteria, with responses categorized as (Yes, No, Unclear, and Not Applicable). Disagreement between reviewers was resolved through discussion, and in cases where consensus was not reached, a third reviewer (ME) was consulted. Each study received a total quality score based on the number of “Yes” responses, with a maximum score of nine. Studies scoring seven or above were considered to have a low risk of bias, while those scoring below seven were categorized as having a high risk of bias. This assessment had no impact on the inclusion or exclusion of the studies.

## Statistical analysis

Meta-analysis was performed using Comprehensive Meta-Analysis software version 4. Both individualized and pooled event rates were calculated, along with 95% CIs for the upper and lower limits, by determining the ratio of the COPD cases to the total sample size. Due to the heterogeneity in the sampling methods and sample sizes across the included studies, we use a random-effect model. Subgroup analyses were performed to explore the prevalence differences according to sex (male vs female), diagnostic criteria for MetS, individual MetS components, and COPD GOLD classification criteria. Sensitivity analysis was performed by analyzing the studies with low risk of bias. Heterogeneity in effect size was examined by calculating the I^2^ index,^49^ to quantify the extent of variability across studies.

## Rresults

### Study selection

**Figure 1.**
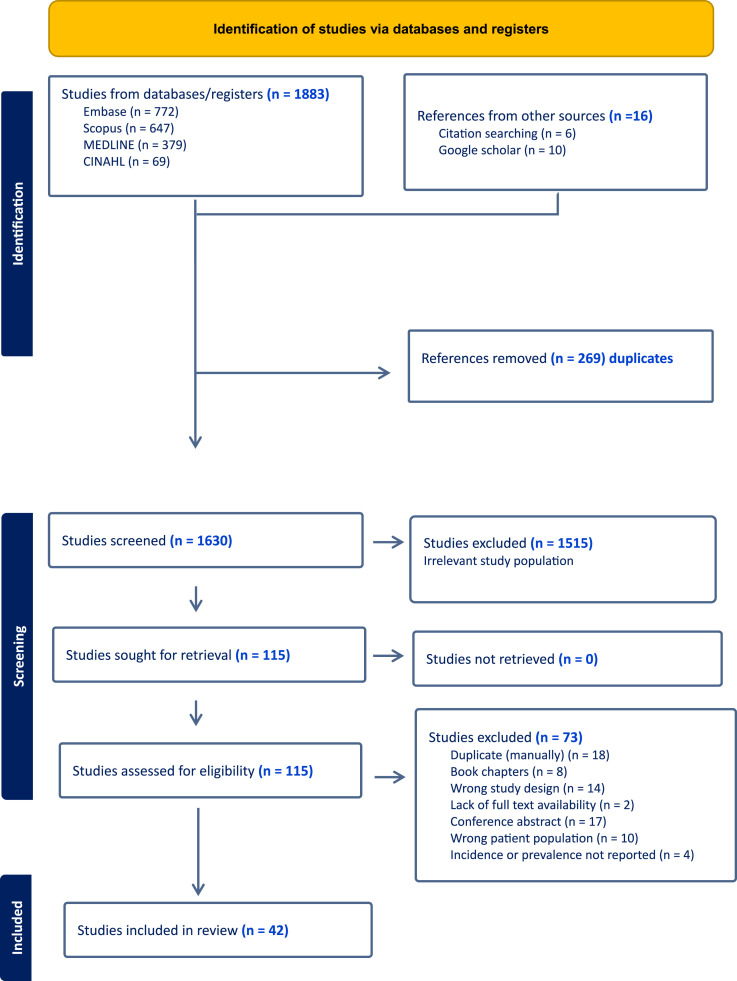
Study screening and selection process for systematic review in accordance with PRISMA.

The database search yielded 1883 citations of potentially relevant studies. After eliminating duplicate entries, 1630 titles and abstracts were reviewed. Out of these, 115 studies were considered relevant and assessed in full text. However, 73 studies were excluded for the following reasons: 18 studies were duplicates identified manually, 8 were book chapters, 14 studies had inappropriate study designs, 2 lacked full-text availability, 17 were conference abstracts, 10 included the wrong patient populations, and 4 did not report prevalence data relevant to MetS (Figure 1). Finally, 42 studies involving 54,278 COPD patients were included in the meta-analysis.

### Characteristics of included studies

A total of 42 articles were included in this study, with sample sizes ranging from 28 to 40,806 participants, providing a comprehensive representation of COPD patients across a diverse population.^38,40–47,50–82^ The severity of COPD was primarily assessed according to the GOLD criteria. However, not all studies provided a detailed breakdown of the distribution across specific GOLD stages (I-IV). Lung function, measured by Forced Expiratory Volume in one second (FEV1 % predicted), was reported in 25 studies, with a pooled mean FEV1 of 60% (SD18.6%), indicating a moderate stage on average. For body mass index (BMI), 33 studies reported values, yielding a pooled mean BMI of 26.2 kg/m^2^ (SD 4.8 kg/m^2^). The age of participants ranged from 53 to 74 years, with a pooled mean of 64.4 years (SD 8.7 years). Regarding sex distribution, male participants comprised between 47.6% and 100% of the study population, with a total of 29,480 male participants (54.3%) and 21,715 female participants (41.1%) across studies. Notably, four studies (9.5%) did not report sex data.^38,44,52,59^ Additionally, only 19 of 42 studies (42.2 %) reported sex-specific prevalence rates for MetS. Detailed sample characteristics are described in supplemental table S3.

Among the 42 studies, 28 studies (67%) employed a cross-sectional design.^38,42,44,45,47,50,52,54,55,57–60,63–67,69,70,73–79,81^ Seven studies (16.6%) used case-control designs, comparing MetS prevalence between COPD and control.^43,51,61,62,68,71,80^ Additionally, four studies (9.5 %) adopted cohort designs, tracking patients over time to assess MetS incidence.^41,53,72,82^The remaining three studies (7.1%) were classified as retrospective, prospective, or observational pilot studies.^40,46,56^ A detailed summary of study designs is reported in supplemental table S3.

### Risk of bias and quality assessment

The quality assessment results are summarized below, with detailed findings provided in Supplemental Table S2. Of the 42 studies, 37 studies (88.6%) achieved a quality score of 7 or higher, indicating a low risk of bias. In contrast, five studies (11.4%) received scores between 5 and 6, reflecting a moderate to high risk of bias.^40,74,75,77,78^ The reliability and generalizability of the findings were directly influenced by study quality. Studies with a low risk of bias were considered more reliable, with stronger prevalence estimates and more representative sampling. These studies generally demonstrated methodological rigor, including a clear sampling method, sufficient sample size, and an appropriately defined study setting. In contrast, studies with moderate to high risk exhibited methodological limitations such as unclear sampling methods and incomplete reporting, reducing the generalizability of their results. Despite these limitations, all studies were included to provide a comprehensive review.

### Geographic Distribution and publication period

India was the most frequently represented country, contributing 10 studies (23.8%).^50,54,59,62,64,65,71,74,75,78^ Other countries with multiple studies included South Korea (4 studies, 9.5%),^44,46,58,79^ Turkey (3 studies, 7.1%),^51,61,63^ and the Netherlands,^53,76^ Italy,^38,40^ Nepal,^52,66^ Canada,^43,80^ and the United States,^47,73^ each contributing 2 studies (4.8%). The remaining studies (35.7%) were distributed across 15 countries, with each contributing a single study: Iran,^57^ Japan,^55^ Germany,^67^ Thailand,^56^ China,^41^ Spain,^70^ Brazil,^82^ UK,^72^ Philippine,^69^ Serbia,^77^ Greece,^42^ Bulgaria,^60^ Hungary,^45^ Slovakia,^81^ and Egypt.^68^

In terms of publication period, three studies (7.1%) were published between 2000 and 2009.^43,67,80^ Most studies, 26 studies (61.9%), were published between 2010 and 2020.^38,40–42,44,46,47,50,51,53,55,58,60–63,68–71,73,74,77,79,81,82^ More recently, 13 studies (30.9%) were published after 2020.^45,52,54,56,57,59,64–66,72,75,76,78^

### Diagnostic criteria for metabolic Syndrome

The studies used various diagnostic criteria for MetS assessment. The National Cholesterol Education Program Adult Treatment Panel III (NCEP ATP-III) criteria were the most frequently used, applied in 21 studies (50%).^40,42,43,46,50,51,54,55,57,59,61,64,65,68,69,72,75,76,78–80^ The International Diabetes Federation (IDF) criteria were used in 16 studies (38.1%).^41,44,45,52,53,56,58,62,63,66,67,71,74,77,81^ The Alberti et al. criteria were used in 6 studies (14.2%).^38,47,60,70,73,82^ Some studies employed more than one diagnostic criterion. Park et al. used both IDF and the American Heart Association/National Heart, Lung, and Blood Institute criteria (AHA/NHLBI),^44^ and Acharyya et al. used both NCEP ATP-III and IDF criteria.^50^

### Pooled prevalence

#### Overall prevalence of (MetS)

The meta-analysis of 42 studies involving 54,278 COPD patients revealed a pooled prevalence of 37% (95% CI 30.6 to 43.8) with high heterogeneity among studies (I^2^ = 99.03%, p < 0.001). These findings are detailed in Table 2, which provides a comprehensive overview of the MetS prevalence across all subgroups.

**Table 2.** Overall prevalence of metabolic syndrome (MetS) stratified by sex, diagnostic criteria, COPD severity, and individual MetS components.

Title	No. of studies	Prevalence rat	95% confidence interval		Heterogeneity (I^2^)	P. Value
			Lower limit	Upper limit		
Overall MetS	42	37.0%	30.6%	43.8%	99.0	0.001
Sex						
Male	19	47.7%	38.1%	57.5%	98.6	0.001
Female	19	43.4%	38.3%	48.8%	87.1	0.001
Diagnostic Criteria						
AHA/NHLBI	1	30.5%	28.8%	32.2%	NA	NA
Alberti	6	45.7%	35.6%	56.3%	90.2	0.001
IDF	16	37.3%	29.8%	45.4%	96.3	0.001
NCEP ATP-III	21	34.6%	25.2%	45.3%	99.2	0.001
COPD severity						
GOLD I	22	26.2%	20.8%	32.4%	97.2	0.001
GOLD II	24	43.7%	37.3%	50.4%	97.8	0.001
GOLD III	23	28.3%	23.5%	33.6%	96.9	0.001
GOLD IV	23	17.8%	10.1%	29.5%	99.4	0.001
MetS Components						
Elevated blood glucose	27	43.0%	31.1%	55.8%	99.6	0.001
Elevated blood pressure	26	58.0%	47.2%	68.0%	99.5	0.001
High Triglyceride	28	36.7%	27.2%	47.4%	99.5	0.001
Increased waist Circumference	28	50.9%	37.1%	64.6%	99.6	0.001
Low HDL	26	35.3%	26.9%	44.7%	99.37	0.001

Abbreviation: MetS, metabolic syndrome; IDF, International Diabetes Federation; NCEP-ATP III, National Cholesterol Education Program’s Third Adult Treatment Panel; AHA/NHLBI, American Heart Association/National Heart, Lung, and Blood Institute; HDL, High-Density Lipoprotein; GOLD, Global Initiative for Chronic Obstructive Lung Disease (GOLD I; Mild COPD, GOLD II; Moderate COPD, GOLD III; Severe COPD, GOLD IV; Very Severe COPD).

Sensitivity analysis, including only studies assessed as low risk of bias,^38,39,41–47,50–69,71,73,76,79–83^ yielded a pooled prevalence of MetS among COPD patients of 37% (95% CI 30 to 44), consistent with the overall estimate. Heterogeneity remained high (I^2^ = 99%).

#### Prevalence of MetS by sex

The prevalence of MetS was reported separately for males and females in 19 studies. The pooled prevalence among male COPD patients was 47.7% (95% CI 38.1 to 57.5) with heterogeneity (I^2^ = 98.64%, p < 0.001). Meanwhile, the prevalence among females was 43.4% (95% CI 38.3 to 48.8), also with substantial heterogeneity (I^2^ = 87.1%, p < 0.001).

#### Prevalence of MetS by diagnostic criteria

The pooled prevalence of MetS varied based on the diagnostic criteria used. Six studies applying the Alberti criteria reported a prevalence of 45.7% (95% CI 35.6 to 56.3), with (I^2^ = 90.18%, p < 0.001). In comparison, 16 studies using the IDF criteria yielded a prevalence of 37.3% (95% CI 29.8 to 45.4), with (I^2^ = 96.30%, p < 0.001). The NCEP ATP-III employed in 21 studies reported a prevalence of 34.6% (95% CI 25.2 to 45.3), accompanied by high heterogeneity (I^2^ = 99.18%, p < 0.001). The AHA/NHLBI criteria were reported in a single study by Park et al.,^44^ with a prevalence rate of 30.5% (95% CI 28.8 to 32.2).

#### Prevalence of MetS by GOLD stages

The prevalence of MetS varied across COPD severity levels based on the GOLD classification. The pooled prevalence was 43.7% (95% CI 37.3 to 50.4) with (I^2^ = 97.84%, p < 0.001) in GOLD II patients. In comparison, GOLD I and GOLD IV had prevalence rates of 26.2% (95% CI 20.8 to 32.4), with (I^2^ = 97.17%, p < 0.001) and 17.8% (95% CI 10.1 to 29.5), with (I^2^ = 99.44%, p < 0.001), respectively. Patients with GOLD III had a prevalence of 28.3% (95% CI 23.5 to 33.6) with (I^2^ = 96.89%, p < 0.001).

#### Prevalence of individual MetS components

Among individual MetS components, hypertension (elevated blood pressure) was the most prevalent, reported in 58% (95% CI 47.2 to 68.0) with (I^2^ = 99.49%, p < 0.001). Increased waist circumference affected 50.9% of patients (95% CI 37.1 to 64.6) with (I^2^ = 99.68%, p < 0.001). Elevated blood glucose was observed in 43% (95% CI 31.1 to 55.8) (I^2^ = 99.61%, p < 0.001), suggesting insulin resistance. High triglycerides were reported in 36.7% (95% CI 27.2 to 47.4) with (I^2^ = 99.49%, p < 0.001), while low HDL cholesterol was observed in 35.3% (95% CI 26.9 to 44.7), with (I^2^ = 99.37%, p < 0.001).

## Discussion

This systematic review and meta-analysis aimed to determine the prevalence of metabolic syndrome among patients with COPD. Our analysis of 54,278 COPD patients from 42 studies revealed a pooled MetS prevalence of 37% (95% CI: 30.6 to 43.8), considerably higher than the global prevalence reported in the general population (12.5% to 31.4 %).^32^ Notably, the most frequently observed components of MetS in the COPD population were hypertension (58%), abdominal obesity (51%), and hyperglycemia (43%). To our knowledge, this is the first comprehensive assessment of the prevalence of MetS and its components in COPD patients. The large sample size supports the observation of an elevated metabolic risk in COPD, with potential implications for increased rates of diabetes, CVD, and premature mortality.^84–86^

Metabolic syndrome is defined by a cluster of conditions, including obesity, dyslipidemia, hypertension, and hyperglycemia, that collectivity increase the risk of T2MD and CVD.^30,87,88^ Our findings highlight the high prevalence of these individual components in COPD patients, underscoring the substantial metabolic burden imposed by the disease.^89,90^ Compared to the general population, COPD patients exhibit higher rates of T2DM and CVD, which are likely attributable to the combined effects of these metabolic abnormalities.^41,56^ Moreover, key pathophysiological features of COPD, including chronic inflammation, oxidative stress, and physical inactivity, further contribute to atherosclerosis and heightened CVD risk.^91,92^ Insulin resistance, a common comorbidity in COPD, also plays a pivotal role in the development of T2DM.^93,94^ Thus, early detection and integrated management of COPD, with particular attention to metabolic abnormalities, are essential for reducing cardiovascular risks, improving quality of life, and lowering healthcare costs.^9,95^

Our analysis indicates that sex does not have a clear impact on the prevalence of MetS in COPD, with a pooled prevalence estimate of 48% in males and 43% in females, with overlapping confidence intervals. Previous studies have reported varying findings regarding the roles of sex and age. For example, Choi et al. observed a higher prevalence of MetS in older females with early-stage COPD compared to their male counterparts.^46^ This observation is supported by studies from Fekete et al. and Kim et al., which attribute the increased prevalence of MetS in females with COPD to inherent biological and hormonal differences, such as reduced estrogen levels, that may lead to increased adiposity and altered lipid metabolism.^45,58^ In contrast, Marquis et al. and Singh et al. reported a higher prevalence in males, suggesting that ethnic background and other population-specific factors may influence these differences.^43,66^ Additionally, age appears to be an influential factor; studies by Dogra et al. and Minas et al. noted a higher prevalence of MetS among younger COPD patients.^42,54^

Beyond sex and age, several studies have highlighted that the presence of comorbidities significantly influences MetS prevalence in COPD. For instance, Karsanji et al., Diez-Manglano et al., and Breyer et al. consistently found that COPD patients with a greater number of comorbidities exhibited a higher prevalence of MetS, emphasizing the complex interplay between metabolic dysfunction and other health conditions.^53,70,72^ These collective findings suggest that while systemic inflammation and oxidative stress are common to both sexes, variations in ethnic background, lifestyle behaviors, and nutritional status may influence individuals' susceptibility to MetS.^43,45,47,66,72^ Moreover, growing evidence suggests that sex may affect the presentation, severity, and outcomes of COPD beyond metabolic parameters. This includes symptom manifestations, disease progression, and treatment response.^83,96,97^

The meta-analysis showed that the use of different diagnostic criteria influenced the prevalence rate for MetS. For instance, prevalence estimates were 31% when using the AHA/NHLBI criteria, 46% with the Alberti criteria, and intermediate values of 37% and 35% with the IDF and NCEP ATP-III criteria, respectively. These variations underline the need for standardized diagnostic approaches to MetS detection. Inconsistencies identified by Park et al. and Acharyya et al.^44,50^ further support the necessity for a uniform set of criteria and perhaps even COPD-specific modification. Given that the pathophysiological features of COPD, such as systemic inflammation, physical inactivity, and coexisting insulin resistance, may alter the presentation of MetS components.^88^ Standard diagnostic thresholds developed for the general population may underestimate or fail to fully capture metabolic risk in COPD patients.^53^ Tailored diagnostic criteria could enhance the accuracy of MetS detection and facilitate earlier intervention in this high-risk group.

A sensitivity analysis was performed to assess whether study quality contributes to the observed heterogeneity, including only studies rated as low risk of bias. The pooled prevalence remains stable at 37% (95% CI 30 to 44), suggesting that factors beyond study quality may contribute.

Geographic region and demographic factors, including age, race, and ethnicity, may contribute to the observed heterogeneity in MetS prevalence across studies.^32,98,99^ For instance, Gurka et al.^99^ highlight that standard MetS criteria might be underestimated in certain populations (such as Asian Americans and non-Hispanic Black males) due to differential risk profiles that are not captured by the existing threshold. Similarly, Asato et al.^98^ demonstrated that genetic predispositions and lifestyle patterns significantly affect metabolic risks across diverse ethnic populations.

However, detailed reporting of race or ethnicity was either inconsistent or absent in many of the included studies in this review. This limitation hinders our ability to conduct stratified analysis or draw conclusions about the influence of ethnicity on MetS prevalence in COPD patients. The absence of this information may also partially explain the observed between-study heterogeneity. We therefore urge future research to adopt standardized demographic reporting, including ethnicity and race, to facilitate more inclusive and representative meta-analysis.

The prevalence of individual MetS components considerably varied across the included studies. Hypertension prevalence ranged from 23%^78^ to 91%,^52^ Triglyceride levels varied from 10%^72^ to 75 %,^70^ and elevated waist circumstances from 22%^70^ to 95%.^45^ These variations likely reflect differences in study methodologies, participant characteristics, and clinical settings (supplemental table S3). Population-specific diagnosis adjustments may improve comparability, and future studies employing standardized diagnostic criteria and controlled study designs are essential to clarify these variations.

There was no clear trend in the prevalence of MetS according to COPD severity, as classified by the GOLD criteria, with rates of 26% in GOLD I, 44% in GOLD II, 28% in GOLD III, and 18% in GOLD IV, with many confidence intervals overlapping. Lifestyle factors such as obesity may impact prevalence. Hacken et al.^100^ identified obesity as a key feature of MetS, particularly in the early stage of COPD, where respiratory symptoms are less severe, potentially contributing to weight gain. In contrast, advanced COPD stages are often characterized by cachexia, a condition involving severe muscle and weight loss.^51,58,77^ This shift may reflect changes in MetS components, such as a reduction in obesity, rather than a true decline in metabolic risk. Future research is needed to identify the interplay between MetS and cachexia in advance of COPD and their respective contributions to disease outcomes.

Interestingly, the obesity paradox in COPD, where higher BMI is associated with lower mortality, adds complexity to our understanding of MetS in this population. While obesity is a defining component of MetS and is generally linked to adverse health outcomes, evidence suggests that in COPD, it may confer a survival advantage.^101,102^ This paradox could imply that the relationship between MetS and COPD outcomes is not straightforward, with obesity potentially offering protective effects that counterbalance the risks posed by other MetS components.^103^ This observation raises questions about how the obesity paradox might influence the interpretation of our findings on MetS prevalence. For instance, if COPD patients with higher BMI are more likely to survive, this could contribute to an elevated prevalence of MetS in cross-sectional studies, given obesity role in the syndrome. However, our analysis did not specifically assess BMI-related mortality outcomes. Future research should explore how the obesity paradox affects the prognostic implication of MetS in COPD, potentially guiding a more tailored clinical approach for these patients.

## Limitations

While this meta-analysis involves a large sample size of 54,278 COPD patients from 42 studies, several limitations warrant consideration. The substantial heterogeneity observed across studies challenges direct result comparison, likely due to various factors. First, the use of different MetS diagnostic criteria (AHA/NHLBI, Alberti, IDF, NCEP ATP-III) introduced variability in reported prevalence rates. Second, variability in study population, including geographic location, age, sex, and inconsistent or absent reporting of race and ethnicity, likely contributed to heterogeneity and limits our ability to explore these variables as sources of variation in MetS prevalence among COPD patients. Third, variations in study methodologies, such as sampling and data collection techniques, may have introduced bias. Lastly, incomplete reporting of sex-specific data and COPD severity limited our ability for detailed subgroup analysis. Future studies should aim for greater demographic diversity and comprehensive data reporting.

## Conclusion

Our findings indicate a higher prevalence of MetS (37%) among COPD patients compared to reported estimates in the general population (12.5 to 31.4%), suggesting an elevated risk of developing CVD, T2DM, and other metabolic complications. These results underscore the need for increased clinical awareness and routine screening for MetS in the COPD population. Tailored screening and management protocols may help identify at-risk individuals, particularly in earlier stages of COPD, where prevalence is higher, potentially guiding more personalized care strategies.

## Supplemental Material

Supplemental Material - The prevalence of metabolic syndrome in chronic obstructive pulmonary disease: A systematic review and meta-analysis Supplemental Material for The prevalence of metabolic syndrome in chronic obstructive pulmonary disease: A systematic review and meta-analysis by Hassan Alrabbaie, Mohammad Al-Wardat, Mohammad Etoom, Marla Beauchamp, Roger Goldstein and Dina Brooks in Chronic Respiratory Disease

## Data Availability

All data generated or analyzed during this study are included in this published article and its supplementary information files.*
