# Supplementary material for: The prevalence of metabolic syndrome in chronic obstructive pulmonary disease: A systematic review and meta-analysis
Source: Chron Respir Dis. 2025 May 26;22:14799731251346194. doi: 10.1177/14799731251346194 (PMC12106998; doi:10.1177/14799731251346194)
Supplement: Supplemental Material - The prevalence of metabolic syndrome in chronic obstructive pulmonary disease: A systematic review and meta-analysis [file sj-pdf-1-crd-10.1177_14799731251346194.pdf]

Table S1: Search Strategy for Identifying the Prevalence of Metabolic Syndrome in Chronic Obstructive Pulmonary Disease, 2000 to June 24, 2024

Ovid MEDLINE:

1- Chronic obstructive pulmonary disease?.mp.
2- Chronic obstructive lung disease?.mp.
3- Chronic obstructive respiratory disease?.mp.
4- COPD.mp.
5- exp Lung Diseases, Obstructive/
6- emphysema.mp. or Emphysema/ or Pulmonary Emphysema/
7- chronic bronchitis.mp. or Bronchitis, Chronic/
8- COAD.mp.
9- ((Chronic Obstructive adj2 disease?) or chronic bronchitis or emphysema).mp. or Bronchitis, Chronic/ or Emphysema/
10- or/1-9
11- ((metabolic or insulin resistance or dysmetabolic or Reaven) adj1 syndrome?).mp.
12- Syndrome x.mp.
13- Metabolic X Syndrome.mp.
14- Dysmetabolic syndrome x.mp
15- Metabolic cardiovascular syndrome.mp
16- or/11-15
17- Prevalences.mp
18- Incidence.mp.
19- (common* or frequen* or comorbid* or multimorbid* or epidemio* or prevalen*).mp.
20- or/17-19
21- 10 and 16 and 20

CINAHL:

1- Chronic obstructive pulmonary disease
2- Chronic obstructive lung disease
3- Chronic obstructive respiratory disease
4- COPD
5- COAD
6- TX (emphysema or Emphysema/ or Pulmonary Emphysema/)
7- TX (chronic bronchitis.mp. or Bronchitis, Chronic/)
8- TX (((Chronic Obstructive adj2 disease?) or chronic bronchitis or emphysema).mp. or Bronchitis, Chronic/ or Emphysema/)
9- S1 OR S2 OR S3 OR S4 OR S5 OR S6 OR S7 OR S8
10- metabolic syndrome or metabolic disease
11- Reaven's syndrome
12- Syndrome X
13- Insulin Resistance
14- Metabolic Syndrome X
15- S10 OR S11 OR S12 OR S13 OR S14
16- prevalence or incidence or epidemiology or frequency or occurrence
17- morbidity
18- epidemiology
19- S16 OR S17 OR S18
20- S9 AND S15 AND S19

Embase:

1- Chronic obstructive pulmonary disease.mp.
2- Chronic obstructive lung disease.mp.
3- Chronic obstructive respiratory disease.mp.
4- COPD.mp.
5- exp "Pulmonary Disease, Chronic Obstructive"/
6- exp "Lung Diseases, Obstructive"/
7- emphysema.mp. or exp Emphysema/ or exp "Pulmonary Emphysema"/
8- "Chronic bronchitis".mp. or exp "Bronchitis, Chronic"/
9- "Chronic Obstructive Airway Disease".mp.
10- COAD.mp.
11- or/1-10
12- Metabolic Syndromes.mp.
13- Insulin Resistance Syndrome.mp.
14- Dysmetabolic Syndrome.mp.
15- Reaven Syndrome.mp.
16- exp Syndrome X/
17- exp Insulin Resistance/
18- MetS.mp.
19- or/12-18
20- Prevalences.mp.
21- Incidence.mp
22- (common* or frequen* or comorbid* or multimorbid* or epidemio* or prevalen*).mp.
23- or/20-22
24- 11 and 19 and 23

Scopus:

1- TITLE-ABS-KEY (("chronic obstructive pulmonary disease*"))
2- TITLE-ABS-KEY ("chronic obstructive lung disease*")
3- TITLE-ABS-KEY ("chronic obstructive respiratory disease*")
4- TITLE-ABS-KEY ("copd")
5- TITLE-ABS-KEY ("chronic obstructive airway disease*")
6- TITLE-ABS-KEY ("emphysema*")
7- TITLE-ABS-KEY ("chronic bronchitis")
8- TITLE-ABS-KEY ("coad")
9- (TITLE-ABS-KEY (("chronic obstructive pulmonary disease*"))) OR (TITLE-ABS-KEY ("chronic obstructive lung disease*")) OR (TITLE-ABS-KEY ("chronic obstructive respiratory disease*")) OR (TITLE-ABS-KEY ("copd")) OR (TITLE-ABS-KEY ("chronic obstructive airway disease*")) OR (TITLE-ABS-KEY ("emphysema*")) OR (TITLE-ABS-KEY ("chronic bronchitis")) OR (TITLE-ABS-KEY ("coad"))
10- TITLE-ABS-KEY ("metabolic syndromes*")
11- TITLE-ABS-KEY ("insulin resistance syndrome*")
12- TITLE-ABS-KEY ("dysmetabolic syndrome*")
13- TITLE-ABS-KEY ("reaven syndrome*")
14- TITLE-ABS-KEY ("metabolic disease*")
15- TITLE-ABS-KEY ("syndrome x")

16- TITLE-ABS-KEY ("mets ")
17- (TITLE-ABS-KEY ("mets ")) OR (TITLE-ABS-KEY ("syndrome x")) OR (TITLE-ABS-KEY ("metabolic disease*")) OR (TITLE-ABS-KEY ("reaven syndrome*")) OR (TITLE-ABS-KEY ("dysmetabolic syndrome*")) OR (TITLE-ABS-KEY ("insulin resistance syndrome*")) OR (TITLE-ABS-KEY ("metabolic syndromes*"))
18- TITLE-ABS-KEY ("prevalence")
19- TITLE-ABS-KEY ("incidence")
20- TITLE-ABS-KEY ("comorbidities")
21- TITLE-ABS-KEY ("frequency")
22- TITLE-ABS-KEY ("epidemiology")
23- (TITLE-ABS-KEY ("epidemiology")) OR (TITLE-ABS-KEY ("frequency")) OR (TITLE-ABS-KEY ("comorbidities")) OR (TITLE-ABS-KEY ("incidence")) OR (TITLE-ABS-KEY ("prevalence"))
24- ((TITLE-ABS-KEY ("epidemiology")) OR (TITLE-ABS-KEY ("frequency")) OR (TITLE-ABS-KEY ("comorbidities")) OR (TITLE-ABS-KEY ("incidence")) OR (TITLE-ABS-KEY ("prevalence"))) AND ((TITLE-ABS-KEY ("mets ")) OR (TITLE-ABS-KEY ("syndrome x")) OR (TITLE-ABS-KEY ("metabolic disease*")) OR (TITLE-ABS-KEY ("reaven syndrome*")) OR (TITLE-ABS-KEY ("dysmetabolic syndrome*")) OR (TITLE-ABS-KEY ("insulin resistance syndrome*")) OR (TITLE-ABS-KEY ("metabolic syndromes*"))) AND ((TITLE-ABS-KEY (("chronic obstructive pulmonary disease*"))) OR (TITLE-ABS-KEY ("chronic obstructive lung disease*")) OR (TITLE-ABS-KEY ("chronic obstructive respiratory disease*")) OR (TITLE-ABS-KEY ("copd")) OR (TITLE-ABS-KEY ("chronic obstructive airway disease*")) OR (TITLE-ABS-KEY ("emphysema*")) OR (TITLE-ABS-KEY ("chronic bronchitis")) OR (TITLE-ABS-KEY ("coad")))

JBI CRITICAL APPRAISAL CHECKLIST for included studies

Q1- Was the sample frame appropriate to address the target population?
Q2- Were study participants sampled in an appropriate way?
Q3 - Was the sample size adequate?
Q4 - Were the study subjects and the setting described in detail?
Q5 - Was the data analysis conducted with sufficient coverage of the identified sample?
Q6 - Were valid methods used for the identification of the condition?
Q7 - Was the condition measured in a standard, reliable way for all participants?
Q8 - Was there appropriate statistical analysis?
Q9 - Was the response rate adequate, and if not, was the low response rate managed appropriately?

	**Author /Year**	**Q1**	**Q2**	**Q3**	**Q4**	**Q5**	**Q6**	**Q7**	**Q8**	**Q9**	**Total Score**
1	Acharyya A, et al. 2016	Yes	Yes	Unclear	Yes	Yes	Yes	Yes	Yes	Unclear	7
2	Akpinar et	Yes	Yes	Unclear	Yes	Yes	Yes	Yes	Yes	Unclear	7

	al.2012										
3	Baniya et al. 2023	Yes	No	Yes	Yes	Yes	Yes	Yes	Yes	Not applicable	7
4	Breyer et al. 2014	Yes	Yes	Yes	Yes	Yes	Yes	Yes	Yes	Not applicable	8
5	Choi et al. 2019	Yes	Yes	Yes	Yes	Yes	Yes	Yes	Yes	Not applicable	8
6	Dogra et al. 2022	Yes	Yes	Yes	Yes	Yes	Yes	Yes	Yes	Not applicable	8
7	Fekete et al. 2022	Yes	Yes	Yes	Yes	Yes	Yes	Yes	Yes	Yes	9
8	Funakoshi et al. 2010	Yes	Yes	Yes	Yes	Yes	Yes	Yes	Yes	Yes	9
9	Keeratichananont et al. 2023	Yes	Yes	Yes	Yes	Yes	Yes	Yes	Yes	Yes	9
10	Kiani et al. 2021	Yes	Yes	Yes	Yes	Yes	Yes	Yes	Yes	yes	9
11	Kim et al. 2019	Yes	Yes	Yes	Yes	Yes	Yes	Yes	Yes	Yes	9
12	Lam et al. 2010	Yes	Yes	Yes	Yes	Yes	Yes	Yes	Yes	Yes	9
13	Mahato et al. 2024	Yes	Yes	Unclear	Yes	Yes	Yes	Yes	Yes	Not applicable	7
14	Marquis et al. 2005	Yes	Yes	Unclear	Yes	Yes	Yes	Yes	Yes	YES	8
15	Mekov, et al. 2015	Yes	Yes	Unclear	Yes	Yes	Yes	Yes	Yes	Yes	8
16	Kupeli et al. 2010	Yes	Yes	Unclear	Yes	Yes	Yes	Yes	Yes	Yes	8
17	Minas et al. 2011	Unclear	Yes	Unclear	Yes	Yes	Yes	Yes	Yes	YES	7
18	Naseem et al. 2019	Yes	Yes	Unclear	Yes	Yes	Yes	Yes	Yes	Not applicable	7
19	Ozgen Alpaydin et al. 2013	Yes	Yes	Unclear	Yes	Yes	Yes	Yes	Yes	Unclear	7
20	Park et al. 2015	Yes	Yes	Yes	Yes	Yes	Yes	Yes	Yes	Not applicable	8
21	Park et al. 2014	Yes	Yes	yes	Yes	Yes	Yes	Yes	Yes	Not applicable	8
22	Piazzolla et al. 2017	Yes	Yes	Unclear	Yes	Yes	Yes	Yes	Yes	Not applicable	7
23	Roy et al. 2022	Yes	Yes	Yes	Yes	Yes	Yes	Yes	Yes	Not applicable	8
24	Sahoo et al. 2022	Yes	Yes	Unclear	Yes	Yes	Yes	Yes	Yes	Not applicable	7
25	Singh et al. 2021	Yes	Yes	Yes	Yes	Yes	Yes	Yes	Yes	Not applicable	8

26	Watz et al. 2009	Yes	Yes	Yes	Yes	Yes	Yes	Yes	Yes	Not applicable	8
27	Ahmed et al. 2020	Yes	Yes	Yes	Yes	Yes	Yes	Yes	Yes	Not applicable	8
28	Bermudez et al. 2018	Yes	Yes	Yes	Yes	Yes	Yes	Yes	Yes	Not applicable	8
29	Dıez-Manglano et al. 2014	Yes	Yes	Yes	Yes	Yes	Yes	Yes	Yes	Not applicable	8
30	Gupta et al. 2017	Yes	Yes	unclear	Yes	Yes	Yes	Yes	Yes	Not applicable	7
31	Karsanji et al. 2022	Yes	Yes	Yes	Yes	Yes	Yes	Yes	Yes	Not applicable	8
32	Park et al. 2014	Yes	Yes	Yes	Yes	Yes	Yes	Yes	Yes	Unclear	8
33	Priyadharshini et al. 2020	unclear	No	Yes	Yes	Yes	Yes	Yes	Yes	Not applicable	6
34	Tiwari et al.2021	unclear	No	No	Yes	Yes	Yes	Yes	Yes	Not applicable	5
35	van Beers et al. 2021	Yes	Unclear	Yes	Yes	Yes	Yes	Yes	Yes	Unclear	7
36	Vujic et al. 2016	Unclear	No	Unclear	Yes	Yes	Yes	Yes	Yes	Not applicable	5
37	Waseem et al. 2021	Unclear	No	Yes	Yes	Yes	Yes	Yes	Yes	Not applicable	6
38	Chung et al. 2015	Yes	Yes	Yes	Yes	Yes	Yes	Yes	Yes	Unclear	8
39	Fumagalli et al. 2013	Unclear	Yes	Unclear	Yes	Yes	Yes	Yes	Yes	Not applicable	6
40	Poulain et al. 2008	Yes	Yes	Unclear	Yes	Yes	Yes	Yes	Yes	Not applicable	7
41	Skyba et al. 2010	Yes	Yes	Unclear	Yes	Yes	Yes	Yes	Yes	Not applicable	7
42	Tanni et al. 2014	Yes	Yes	Unclear	Yes	Yes	Yes	Yes	Yes	Not applicable	7

S3: Characteristics of COPD patients and the prevalence of metabolic syndrome and its components in included studies

Author /Year	Population (setting)	COPD Diagnostic Method	Country	Study type	Number of COPD Patients	Number of Male (%)	Number of Female (%)	Mean Age/SD	BMI (kg/m²) mean/SD	Prevalence MetS GOLD (%)				FEV1, % pred	Definition /Criteria	Overall MetS Prevalence (%)	Male MetS Prevalence (%)	Female MetS Prevalence (%)	Prevalence of MetS Components (%)				
										I	II	III	IV						WC	BG	BP	TG	HDL
Acharyya A, et al. 2016 (1)	COPD Patients from The Department of Pulmonary Medicine	Spirometry test and hyperinflation in chest X-ray	India	Cross-sectional study	77	57 (74)	20 (26)	60 ±10	23± 6	-	-	-	-	-	NCEP ATP-III/IDF	44.15/31.16	-	-	-	-	-	-	-
Akpinar et	Outpatients	GOLD	Turkey	case-control	91	78 (86)	13 (14)	64 ± 9	30±6	38.5	52.8	30	33.3	-	NCEP	44.6	-	-	52.2	46.7	77.2	25	34.8

al.2012 (2)	with stable COPD	criteria - Spirometry test		study											ATP- III								
Baniya et al. 2023 (3)	Outpatients with Stable COPD	Pulmonary clinics – GOLD criteria	Nepal	Descriptive Cross-sectional Study	57	-	-	69±8	29 ± 2	27.3	40.9	27.3	4.5	-	IDF	38.6	-	-	38.6	86.4	90.9	31.8	40.9
Breyer et al. 2014 (4)	Clinically stable COPD as part of the CIROCO study	GOLD criteria- Spirometry test	Netherlands	Prospective cohort study	228	134 (59)	94 (41)	64 ± 7	26± 5	8	50	31	13	53 ± 19	IDF	57	-	-	79	49	47	31	10
Choi et al. 2019 (5)	Patients with early COPD KNHANES database	GOLD criteria- Spirometry test	South Korea	Retrospective observational study	2164	1566 (72)	598 (28)	65±10	24±3	26.8	25.9	23.4	23.9	88±14	NCEP ATP- III	31.2	26.7	35.1	29.8	12.8	59.7	39.8	47.3
Dogra et al. 2022 (6)	Outpatients with Stable COPD	GOLD criteria- spirometry test	India	Cross-sectional study	100	93 (93)	7 (7)	60 ± 9	24±6	6.6	28.8	42.2	22.2	50±19	NCEP ATP- III	45	-	-	45	37	38	34	27
Fekete et al. 2022 (7)	Stable COPD patients from the Pulmonary Rehabilitation ward	GOLD criteria- validated self-reported	Hungary	Cross-sectional study	401	191 (48)	210 (52)	67±10	-	56.7	65	60.4	46.4	-	IDF	59.4	40.1	59.9	95.4	79.3	89.8	48.5	38.8
Funakoshi et al. 2010 (8)	Patients underwent a comprehensive health screening	GOLD criteria- Spirometry test	Japan	Cross-sectional study	645	645 (100)	0	62 ± 9	23±3	-	25.8	16.8	28.7	-	NCEP ATP- III	23	-	-	24	54	52	33	5
Keeratichananont et al. 2023 (9)	Outpatients' stable COPD at a tertiary hospital that is a major referral center for 14 provinces in the southern Thailand	GOLD criteria - Spirometry test	Thailand	Prospective observational study	115	110 (90)	11 (10)	73± 7	30 ± 3	18.6	27.9	23.3	30.2	67±20	IDF	37.4	95.3	4.7	-	30.2	25.6	30.2	27.9
Kiani et al. 2021 (10)	Part of the PERSIAN (Prospective Epidemiological Research Studies in Iran) cohort study	GOLD critera- Spirometry test	Iran	Population-based cross-sectional study	215	120 (56)	95 (44)	53± 10	28± 5	-	-	-	-	79 ± 18	NCEP ATP- III	22.8	19.2	27.4	-	-	-	-	-
Kim et al. 2019 (11)	Secondary analysis of population-based data obtained from the KNHANES -V nationwide health	GOLD criteria - Spirometry test	South Korea	Cross-sectional study	1237	903 (73)	334 (27)	65± 10	24± 3	-	-	-	-	-	IDF	23	18.5	38.5	35	23	63	39	49

	survey.																						
Lam et al. 2010 (12)	Patients with airway obstruction from the Guangzhou Health and Happiness Association for Respectable Elders (GHHARE)	GOLD criteria- Spirometry test	China	Population-based cohort study	496	128(26)	368(74)	64 ±6	-	-	-	-	-	-	IDF	23	-	-	34.1	34.3	56.7	29.6	15.9
Mahato et al. 2024 (13)	Patients with COPD visiting the Index Medical College and Hospital.	GOLD criteria – Spirometry test	India	Cross-sectional study	100	-	-	-	27 ± 6	-	-	-	-	-	NCEP ATP-III	44	-	-	45	52	-	45	48
Marquis et al. 2005 (14)	Stable COPD patients entered a 12-week cardiopulmonary rehabilitation program	Clinical evaluation – Pulmonary function test and Arterial blood gases	Canada	Case-control study	38	23 (61)	15 (39)	66±7	28±5	-	-	-	-	63 ±16	NCEP APT- III	47	61	27	61	13	82	63	24
Mekov, et al. 2015 (15)	COPD patients admitted for exacerbation were recruited from university-specialized hospitals for active treatment of pulmonary diseases	GOLD criteria – Spirometry test	Bulgaria	Cross-sectional study	152	108 (71)	44(29)	65±10	27	-	-	-	-	55±20	Alberti	25	23.1	29.5	28.3	34.2	69.1	29.6	15.8
Kupeli et al. 2010 (16)	COPD patients regularly follow up at the Pulmonary Diseases Department	GOLD criteria- Spirometry test	Turkey	Prospective case-control study	106	92 (87)	14 (13)	67 ± 9	28±6	34	34	24	7	63 ±24	NCEP APT- III	27	82.8	17.2	-	-	-	-	-
Minas et al. 2011 (17)	Patients with COPD from outpatients with mild to severe airflow limitation (GOLD stage I to IV)	GOLD criteria- Spirometry test	Greece	Cross-sectional study	114	114(100)	0	66 (62±71) *	26 ± 5	16.7	66.7	-	-	64 (53 ±68) *	NCEP ATP- III	21	-	-	28.1	24.6	25.4	22.8	16.7
Naseem et al. 2019 (18)	An outpatient with COPD	GOLD criteria - spirometry	India	Observational Case-Control Study	150	109(72)	41 (28)	59±7	-	75	32	17	13.5	49±19	IDF	27.3	-	-	-	-	-	-	-

	attending Pulmonology Department	test																					
Ozgen Alpaydin et al. 2013 (19)	COPD patients referred to the pulmonary disease' outpatient clinic	GOLD criteria – spirometry	Turkey	Cross-sectional study	50	45 (90)	5 (10)	61 ± 6	27 ± 5	33	72	33	12	46 ± 17	IDF	44	-	-	-	-	-	-	-
Park et al. 2015 (20)	Patients participated in the health checkup program at the Health Promotion Center of Samsung Medical Center	CT scan – spirometry test	South Korea	Retrospective cross-sectional study	2814	-	-	55 ± 8	-	-	-	-	-	87 ± 13	IDF, (AHA/NHLBI)	21.6 / 30.5	-	-	35.3	37.6	45.7	40	23.4
Park et al. 2014 (21)	National Health and Nutrition Examination Survey (NHANES), patients diagnosed with emphysema or chronic bronchitis	Physician diagnoses	United States	Secondary analysis of cross-sectional data	223	114 (51)	109 (49)	70±9	29±7	-	-	-	-	-	Alberti	55	-	-	66.7	66.4	79	72.2	51
Piazzolla et al. 2017 (22)	COPD patients attending the outpatient clinic of the Pneumonology Department	GOLD criteria-Spirometry test	Italy	Cross-sectional study	76	-	-	67 ± 9	31 ± 5	57.9	22.8	3.5	15.8	69 ± 20	Alberti	62	-	-	-	-	-	-	-
Roy et al. 2022 (23)	In and outpatients with COPD	GOLD criteria-Spirometry test and X-Ray	India	Cross-sectional study	210	0	210(100)	63±10	22±5	11.4	27.1	17.1	4.8	-	NCEP ATP- III	60.5	-	-	-	-	-	-	-
Sahoo et al. 2022 (24)	COPD from outpatient or getting Admitted to the Department of Pulmonary Medicine	GOLD criteria-Pulmonary function test and clinical examination	India	Cross-sectional observational study	76	59 (78)	17(22)	63 ± 9	24±4	3.1	31.2	59.4	6.3	48±12	NCEP ATP- III	42.1	33.9	70.6	28.9	39.5	59.2	36.8	38.1
Singh et al. 2021 (25)	COPD patients in a stable clinical condition	GOLD criteria-Pulmonary function test and clinical examination	Nepal	Descriptive cross-sectional study	84	35 (42)	49 (58)	67±11	-	50	38	30	28.6	-	IDF for South Asians.	35.7	40	32.65	35.7	11.9	23.8	39.3	33.3

Watz et al. 2009 (26)	Stable outpatients with COPD of different levels of severity	GOLD criteria-Spirometry test	Germany	Cross-sectional study	170	151 (76)	19 (24)	64 +7	26± 5	50	53	37	44	63 ±26	IDF	47.5			78	38	75	37	31
Ahmed et al. 2020 (27)	Patients with stable COPD from the chest clinic	GOLD criteria-Spirometry test	Egypt	Case-control study	430	397 (92)	33 (8)	58±8	-	0	39.2	43.9	43	-	NCEP ATP- III	41.6	39	72.7	35.6	31.6	34.4	32	32
Bermudez et al. 2018 (28)	COPD patients attending the Pulmonary Medicine outpatient clinic	GOLD criteria-Spirometry test and clinical examination	Philippines	Cross-sectional analytic study	157	127 (81)	30 (19)	64±9	21± 4	31.8	52.4	9.8	6.4	-	NCEP ATP- III	40.1	-	-	23	39	46	19	36
Dıez-Manglano et al. 2014 (29)	Hospitalized patients for COPD exacerbation	GOLD criteria-Spirometry test	Spain	Observational, cross-sectional	375	333 (89)	42 (11)	74± 9	27± 5	-	40.4	52.2	7.4	43±12	Alberti	42.9	40.8	59.5	22	58	70	53	13
Gupta et al. 2017 (30)	Patients with COPD attending the outdoor and indoor general medicine department	GOLD criteria-Spirometry test	India	Analytical Case-Control Study	90	62 (69)	28 (31)	53±7	23±3	-	-	-	-	-	IDF	33.3	80	20	78.9	18.8	26.7	20	25.5
Karsanji et al. 2022 (31)	Patients with COPD from the Clinical Practice Research Datalink (CPRDGOLD) between 2009 to 2017	GOLD criteria-Spirometry test	UK	Retrospective longitudinal cohort study	40806	22157 (54)	18649 (46)	69±11	28±6	20.2	54.3	22.6	2.9	63±23	NCEP ATP- III	10.1	58.46	41.54	84.3	78.6	84.9	10.1	69.1
Park et al. 2014 (32)	National Health and Nutrition Examination Survey (NHANES), patients diagnosed with emphysema or chronic bronchitis	GOLD criteria-Spirometry test	United States	Cross-sectional study	94	42 (45)	52(55)	62± 10	27±6	-	-	-	-	67±21	Alberti	57.5	50	50	53	68	66	49	52
Priyadharshini et al. 2020 (33)	Patients clinically diagnosed with any stage of COPD from the unit of Pulmonology	GOLD criteria-Spirometry test	India	Cross-sectional study	76	76 (100)	0	63 ± 11	-	-	-	-	-	-	IDF	54	-	-	70	-	-	-	-

Tiwari et al.2021 (34)	COPD patients from the Chest Department	GOLD criteria-Spirometry test	India	Cross-sectional study	62	34 (55)	28 (45)	64±10	-	17.2	55	20.7	6.9	-	NCEP ATP- III	46.8	-	-	-	-	-	-	-
van Beers et al. 2021 (35)	Patients with COPD referred for Pulmonary Rehabilitation	GOLD criteria-Lung function test	Netherlands	Cross-sectional study	170	91 (54)	79 (46)	63 ± 9	27 ±6	-	-	-	-	55 ±23	NCEP ATP-III	54.7	57.1	51.9	-	-	-	-	-
Vujic et al. 2016 (36)	Outpatient COPD at the Clinic for Pulmonology	GOLD criteria-Spirometry test	Serbia	Cross-sectional study	98	63 (64)	35 (36)	63 ± 7	25 ±5	33.3	48.8	31.6	23.1	39 ±15	IDF	37.8	56.75	45.7	-	-	-	-	-
Waseem et al. 2021 (37)	COPD patients from the tuberculosis and respiratory diseases outpatient department	GOLD criteria-Spirometry test	India	Cross-sectional study	336	195 (58)	141 (42)	-	-	24.2	32	17.5	19	-	NCEP ATP-III	26.5	41.57	58.43	80.9	65.2	22.5	70.8	84.3
Chung et al. 2015 (38)	National Health and Nutrition Examination Survey (KNHANES)	GOLD criteria – Spirometry test	South Korea	Cross-sectional study	1039	760 (73)	279 (27)	65 ± 10	24 ± 3	-	-	-	-	77±16	NCEP ATP-III	32	29.5	38	-	-	-	-	-
Fumagalli et al. 2013 (39)	COPD Outpatient in four major hospitals in Rome	GOLD criteria-Spirometry test and radiological exam	Italy	Observational pilot study	169	124 (73)	45 (27)	74 ± 8	27 ± 5	-	-	-	-	56 ±20	NCEP ATP-III	21	-	-	-	-	-	-	-
Poulain et al. 2008 (40)	COPD patients engaged in the pulmonary rehabilitation program	GOLD criteria – Pulmonary function test	Canada	Case-control study	28	28 (100)	0	65 ± 5	28 ± 4	-	-	-	-	42 ±16	NCEP ATP-III	29	-	-	-	-	-	-	-
Skyba et al. 2010 (41)	Outpatients diagnosed with COPD , free from exacerbation for ≥8 weeks	GOLD criteria– Spirometry test	Slovakia	Cross-sectional study	44	38 (86)	6 (14)	62 ± 7	27± 7	-	-	-	-	54 ± 23	IDF	39	-	-	66	39	57	20	9
Tanni et al. 2014 (42)	Patients with mild to very severe COPD	GOLD criteria – Spirometry test	Brazil	Prospective Cohort Study	115	78 (68)	37 (32)	65 ± 10	26± 6	-	-	-	-	59 ± 25	Alberti	35.6	-	-	33	-	48	33	-

Abbreviations: MetS , Metabolic Syndrome; COPD, Chronic Obstructive Pulmonary Disease; SD, Standard Deviation, BMI, Body Mass Index, GOLD, Global Initiative for Obstructive Lung Disease; FEV1, Forced Expiratory Volume in 1 second; WC, Waist Circumference; BG, Blood Glucose; BP, Blood Pressure; TG, Triglycerides; HDL, High-Density Lipoprotein; IDF, International Diabetes Federation criteria (2005); NCEP: ATPIII National Cholesterol Education Program Adult Treatment Panel III criteria (2001); Alberti American Heart Association/National Heart, Lung, and Blood Institute (AHA/NHLBI) criteria (2004).
*Median (Interquartile rang)

References:

1. Acharyya A, Shahjahan MD, Mesbah FB, Dey SK, Ali L. Association of metabolic syndrome with chronic obstructive pulmonary disease in an Indian population. Lung India. 2016;33(4):385-90.
2. Akpinar EE, Akpinar S, Ertek S, Sayin E, Gülhan M. Systemic inflammation and metabolic syndrome in stable COPD patients. Tuberk. 2012;60(3):230-7.
3. Baniya S, Shrestha TM, Pant P, Aacharya RP. Metabolic syndrome among stable chronic obstructive pulmonary disease patients visiting outpatient department of a tertiary care centre: A descriptive cross-sectional study. JNMA: Journal of the Nepal Medical Association. 2023;61(260):355.
4. Breyer MK, Spruit MA, Hanson CK, Franssen FME, Vanfleteren LEGW, Groenen MTJ, et al. Prevalence of metabolic syndrome in COPD patients and its consequences. PLoS ONE. 2014;9(6).
5. Choi HS, Rhee CK, Park YB, Yoo KH, Lim SY. Metabolic syndrome in early chronic obstructiv pulmonary disease: Gender differences and impact on exacerbation and medical costs. Int J Chron Obstruct Pulmon Dis. 2019;14:2873-83.
6. Dogra M, Jaggi S, Aggarwal D, Gupta S, Saini V, Kaur J. Role of interleukin-6 and insulin resistance as screening markers for metabolic syndrome in patients of chronic obstructive pulmonary disease. A hospital-based cross-sectional study. Monaldi Arch Chest Dis. 2022;92(3).
7. Fekete M, Szollosi G, Tarantini S, Lehoczki A, Nemeth AN, Bodola C, et al. Metabolic syndrome in patients with COPD: Causes and pathophysiological consequences. Physiol. 2022;109(1):90-105.
8. Funakoshi Y, Omori H, Mihara S, Marubayashi T, Katoh T. Association between airflow obstruction and the metabolic syndrome or its components in Japanese men. Intern Med. 2010;49(19):2093-9.
9. Keeratichananont W, Kaenmuang P, Geater SL, Manoret P, Thanapattaraborisuth B. Prevalence, associated factors, and clinical consequences of metabolic syndrome in chronic obstructive pulmonary disease patients: a 5-year prospective observational study. Therap. 2023;17:17534666231167342.
10. Kiani FZ, Ahmadi A. Prevalence of different comorbidities in chronic obstructive pulmonary disease among Shahrekord PERSIAN cohort study in southwest Iran. Scientific reports. 2021;11(1):1548.
11. Kim J, Yoo JY, Kim HS. Metabolic Syndrome in South Korean Patients with Chronic Obstructive Pulmonary Disease: A Focus on Gender Differences. Asian Nursing Research. 2019;13(2):137-46.
12. Lam KBH, Jordan RE, Jiang CQ, Thomas GN, Miller MR, Zhang WS, et al. Airflow obstruction and metabolic syndrome: The Guangzhou Biobank Cohort Study. European Respiratory Journal. 2010;35(2):317-23.
13. Mahato B, Nigoskar S, Lakshmi LJ, Zephy D. Glycemic Profile and Lipid Profile in Chronic Obstructive Pulmonary Disease (COPD) Patients With and Without Metabolic Syndrome. Cureus. 2024;16(4):e58921.
14. Marquis K, Maltais F, Duguay V, Bezeau AM, LeBlanc P, Jobin J, et al. The metabolic syndrome in patients with chronic obstructive pulmonary disease. Journal of Cardiopulmonary Rehabilitation. 2005;25(4):226-32.
15. Mekov E, Slavova Y, Tsakova A, Genova M, Kostadinov D, Minchev D, et al. Metabolic syndrome in hospitalized patients with chronic obstructive pulmonary disease. Peerj. 2015;2015(7).

16. Küpeli E, Ulubay G, Ulasli SS, Sahin T, Erayman Z, Gürsoy A. Metabolic Syndrome is associated with increased risk of acute exacerbation of COPD: A preliminary study. Endocrine. 2010;38(1):76-82.
17. Minas M, Kostikas K, Papaioannou AI, Mystridou P, Karetsi E, Georgoulias P, et al. The association of metabolic syndrome with adipose tissue hormones and insulin resistance in patients with COPD without Co-morbidities. COPD: Journal of Chronic Obstructive Pulmonary Disease. 2011;8(6):414-20.
18. Naseem S, Baneen U. Systemic inflammation in patients of chronic obstructive pulmonary disease with metabolic syndrome. Journal of family medicine and primary care. 2019;8(10):3393-8.
19. Ozgen Alpaydin A, Konyar Arslan I, Serter S, Sakar Coskun A, Celik P, Taneli F, et al. Metabolic syndrome and carotid intima-media thickness in chronic obstructive pulmonary disease. Multidiscip. 2013;8(1):61.
20. Park HY, Jhun BW, Jeong HJ, Chon HR, Koh WJ, Suh GY, et al. The complex association of metabolic syndrome and its components with computed tomography-determined emphysema index. Metabolic Syndrome and Related Disorders. 2015;13(3):132-9.
21. Park SK, Larson JL. The relationship between physical activity and metabolic syndrome in people with chronic obstructive pulmonary disease. Journal of Cardiovascular Nursing. 2014;29(6):499-507.
22. Piazzolla G, Castrovilli A, Liotino V, Vulpi MR, Fanelli M, Mazzocca A, et al. Metabolic syndrome and Chronic Obstructive Pulmonary Disease (COPD): The interplay among smoking, insulin resistance and vitamin D. PLoS ONE. 2017;12(10).
23. Roy R, Gautam AK, Singh NP, Kumar A. Metabolic syndrome and its correlates among female chronic obstructive pulmonary disease patients at a rural tertiary health care center in Northern India. Cureus. 2022;14(8).
24. Sahoo KC, Subhankar S, Mohanta PC, Jagaty SK, Dutta P, Pothal S. Prevalence of metabolic syndrome in chronic obstructive pulmonary disease and its correlation with severity of disease. J. 2022;11(5):2094-8.
25. Singh NK, Karki L. Metabolic syndrome in patients with chronic obstructive pulmonary disease in medicine department of a tertiary care hospital: A descriptive cross-sectional study. JNMA: Journal of the Nepal Medical Association. 2021;59(236):313.
26. Watz H, Waschki B, Kirsten A, Müller KC, Kretschmar G, Meyer T, et al. The metabolic syndrome in patients with chronic bronchitis and COPD: frequency and associated consequences for systemic inflammation and physical inactivity. CHEST. 2009;136(4):1039-46.
27. Ahmed MES, Elnaby HEHA, Hussein MAR, Abo-Ghabsha ME. Metabolic syndrome in patients with chronic obstructive pulmonary disease. Egyptian Journal of Chest Diseases and Tuberculosis. 2020;69(2):316-22.
28. Bermudez GR, Jasul G, David-Wang A, Jimeno C, Magallanes J, Macalalad-Josue AA. Association of metabolic syndrome with the severity of airflow obstruction in patients with chronic obstructive pulmonary disease. J. 2018;33(2):181-7.
29. Diez-Manglano J, Barquero-Romero J, Almagro P, Cabrera FJ, Lopez Garcia F, Montero L, et al. COPD patients with and without metabolic syndrome: clinical and functional differences. Internal and emergency medicine. 2014;9(4):419-25.
30. Gupta KK, Singh J, Gupta P, Patel ML, Kumar V, Chaudhary SC. Uncovering metabolic syndrome among chronic obstructive pulmonary disease patients in a tertiary care hospital, India. Journal of Clinical and Diagnostic Research. 2017;11(5):OC08-OC11.
31. Karsanji U, Evans RA, Quint JK, Khunti K, Lawson CA, Petherick E, et al. Mortality associated with metabolic syndrome in people with COPD managed in primary care. ERJ open res. 2022;8(4).
32. Park SK, Larson JL. Metabolic syndrome and associated factors in people with chronic obstructive pulmonary disease. Western Journal of Nursing Research. 2014;36(5):620-42.

33. Priyadharshini N, Renusha RC, Reshma S, Sindhuri Sai M, Koushik Muthu RM, Rajanandh MG. Prevalence of metabolic syndrome in patients with chronic obstructive pulmonary disease: An observational study in South Indians. Diabetes and Metabolic Syndrome: Clinical Research and Reviews. 2020;14(4):503-7.
34. Tiwari A, Piruwa SK, Agrawaal SK, Srivastava GN, Shah D. Prevalence of metabolic syndrome in copd in rural population of developing country- a cross-sectional study. Journal of Clinical and Diagnostic Research. 2021;15(3):OC30-OC3.
35. van Beers M, Gosker HR, Janssen DJA, Cleutjens FAHM, Franssen FME, van Boxtel MPJ, et al. Cognitive performance in relation to metabolic disturbances in patients with COPD. Clinical Nutrition. 2021;40(4):2061-7.
36. Vujic T, Nagorni Obradovic L, Maric G, Popovic L, Jankovic J. Metabolic syndrome in patients with chronic obstructive pulmonary disease: Frequency and relationship with systemic inflammation. Hippokratia. 2016;20(2):110-4.
37. Waseem SMA, Islam N. A study of metabolic syndrome in chronic obstructive pulmonary disease patients attending out-patient department of a medical college. Physiology and Pharmacology. 2021;25(2):108-15.
38. Chung JH, Hwang H-J, Han CH, Son BS, Kim DH, Park MS. Association between sarcopenia and metabolic syndrome in chronic obstructive pulmonary disease: the Korea National Health and Nutrition Examination Survey (KNHANES) from 2008 to 2011. COPD: Journal of Chronic Obstructive Pulmonary Disease. 2015;12(1):82-9.
39. Fumagalli G, Fabiani F, Forte S, Napolitano M, Marinelli P, Palange P, et al. INDACO project: A pilot study on incidence of comorbidities in COPD patients referred to pneumology units. Multidiscip. 2013;8(4).
40. Poulain M, Doucet M, Drapeau V, Fournier G, Tremblay A, Poirier P, et al. Metabolic and inflammatory profile in obese patients with chronic obstructive pulmonary disease. Chronic Respiratory Disease. 2008;5(1):35-41.
41. Skyba P, Ukropec J, Pobeha P, Ukropcova B, Joppa P, Kurdiova T, et al. Metabolic phenotype and adipose tissue inflammation in patients with chronic obstructive pulmonary disease. Mediators Inflamm. 2010;2010(1):173498.
42. Tanni SE, Zamuner AT, Coelho LS, Vale SA, Godoy I, Paiva SA. Are metabolic syndrome and its components associated with 5-year mortality in chronic obstructive pupmonary disease patients? Metab. 2015;13(1):52-4.
